# Accurate Determination of the *Q* Quality Factor in Magnetoelastic Resonant Platforms for Advanced Biological Detection

**DOI:** 10.3390/s18030887

**Published:** 2018-03-16

**Authors:** Ana Catarina Lopes, Ariane Sagasti, Andoni Lasheras, Virginia Muto, Jon Gutiérrez, Dimitris Kouzoudis, José Manuel Barandiarán

**Affiliations:** 1 BCMaterials, Bld. Martina Casiano, 3rd Floor, UPV/EHU Science Park, Barrio Sarriena s/n, 48940 Leioa, Spain; catarina.lopes@bcmaterials.net (A.C.L.); jon.gutierrez@ehu.eus (J.G.); manu@bcmaterials.net (J.M.B.); 2 Departamento de Matemática Aplicada, Universidad del País Vasco UPV/EHU, Torres Quevedo 1, C.P., 48013 Bilbao, Spain; andoni.lasheras@ehu.eus; 3 Departamento de Matemática Aplicada y Estadística e Investigación Operativa, Universidad del País Vasco UPV/EHU, P.O. Box 644, 48080 Bilbao, Spain; virginia.muto@ehu.eus; 4 Departamento de Electricidad y Electrónica, Universidad del País Vasco UPV/EHU, P.O. Box 644, 48080 Bilbao, Spain; 5 Department of Chemical Engineering, University of Patras, 26504 Patras, Greece; kouzoudi@upatras.gr

**Keywords:** magnetic biosensors, quality factor, magnetoelastic resonance

## Abstract

The main parameters of magnetoelastic resonators in the detection of chemical (i.e., salts, gases, etc.) or biological (i.e., bacteria, phages, etc.) agents are the sensitivity S (or external agent change magnitude per Hz change in the resonance frequency) and the quality factor Q of the resonance. We present an extensive study on the experimental determination of the Q factor in such magnetoelastic resonant platforms, using three different strategies: (a) analyzing the real and imaginary components of the susceptibility at resonance; (b) numerical fitting of the modulus of the susceptibility; (c) using an exact mathematical expression for the real part of the susceptibility. Q values obtained by the three methods are analyzed and discussed, aiming to establish the most adequate one to accurately determine the quality factor of the magnetoelastic resonance.

## 1. Introduction

Magnetoelastic resonators used as sensing devices present advantages like allowing remote “query and answer” [1,2] as well as low cost and low power consumption [3]. Due to these reasons, chemical and many other parameters can be detected: aqueous chemicals including pH [1], salt, and glucose concentrations [3], as well as inorganic salt depositions [4], gas humidity [5], gases such as carbon dioxide [6], or toxic volatile organic compounds (VOCs) such as benzene or hexane, among others [7]. In recent years, they have become a hot topic as novel wireless biosensors for bacteria, potentially lethal for humans, such as *Salmonella* [8,9], *Bacillus anthracis* [9], or *Escherichia coli* [10]. Such detection will be achieved if the surface of the magnetoelastic resonator is coated with an appropriate smart functionalized film that interacts with the target of interest.

**Figure 1:**
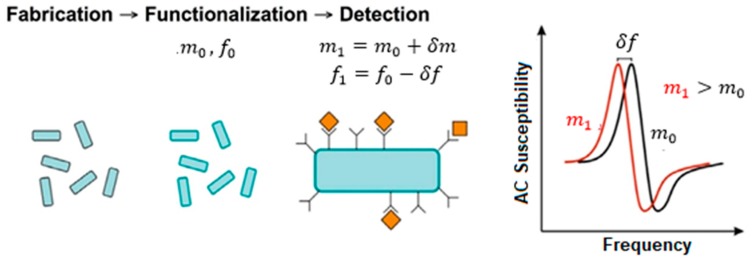
Principle of detection of biological targets using magnetoelastic resonators. The adhesion of the bacteria to the materials leads to an increase in the total mass of the system, which is detected as a shift (always decreasing) in the measured magnetoelastic resonance frequency.

The detection process in such sensors is based on the shift of the magnetoelastic resonance (MER) frequency under the action of an external agent, easily seen when measuring the magnetic susceptibility versus the frequency of the applied magnetic field. In the case of biological agents, the adhesion of different bacteria to the resonators causes an increase in the total mass, which leads to a decrease in the MER (see Figure 1).

Among the magnetoelastic materials to be used as biological sensors, Fe-based amorphous ferromagnetic alloys in the form of a ribbon are among the most suitable, mainly due to their high magnetoelastic coupling coefficient (k), high saturation magnetostriction ($\lambda_{S}$), and high saturation magnetization ($M_{S}$) [11].

The good performance of a magnetoelastic sensing device is mainly determined by two parameters: sensitivity and quality factor. The sensitivity is related to the lowest detectable frequency change. This depends on the experimental system, but also on the sharpness of the resonance, which in turns depends on the Q factor. In some cases, the determination of Q can be more sensitive than that of the resonance frequency for detecting small changes in mass. With $m_{0}$ and $f_{0}$ the unloaded mass and corresponding MER frequency of a magnetoelastic resonator, respectively, its sensitivity to a change in mass due to an external target is given by the relationship:

(1) $$S=-\frac{\delta f}{\delta m}=\frac{f_{0}}{2m_{0}},$$

where δf represents the resonance frequency shift caused by the presence of an external agent that causes a change of mass δm, mass change of the MER film. Thus, a high sensitivity value means a large δf shift for a given mass change. The linearity expressed by Equation (1) is valid for small mass changes compared to the initial MER film mass. Nevertheless, it is just an approximation of a more general expression [12] and is still subject to revision and discussion by the authors [13,14].

Concerning the quality factor Q, it has been already experimentally observed that damping strongly affects both resonant frequency and magnetoelastic resonance curve shape (see, for example, [15,16,17]). A high Q value means a sharp resonance frequency and, consequently, a well-defined $f_{r}$ resonance frequency. From the measured susceptibility curve around the magnetoelastic resonance, the Q quality factor can be estimated as the resonance curve full bandwidth Δf signal (or full width at half maximum power) relative to its susceptibility maximum frequency $f_{r}$, that is:

(2) $$Q_{0}=\frac{f_{r}}{\Delta f},$$

which is a dimensionless number [18,19]. This classical empirical first approximation can lead to errors as high as 20% in the correct Q value determination, as previously noted by Kaczkowski [20]. Therefore, in biological detection based on the magnetoelastic resonance frequency shift, accurate determination of the Q quality factor beyond the empirical expression Equation (2) turns out to be a key parameter.

In the present work, we present an extensive study of the determination of the Q factor in magnetoelastic resonant platforms. To do this, strips (L=4 cm) of Fe-rich $Fe_{64}Co_{17}Si_{6.6}B_{12.4}$ composition homemade metallic glass have been used. Determination of the Q quality factor value has been performed in three different ways: (a) analyzing the full susceptibility curve around the resonance (real and imaginary components); (b) numerical fitting of the magnitude (modulus) of the susceptibility; and (c) using an exact mathematical expression for the Q value arising from analysis of the real part of the susceptibility curve at the resonance.

## 2. Experimental

### 2.1. Material: Magnetic and Magnetostrictive Characterization

**Figure 2:**
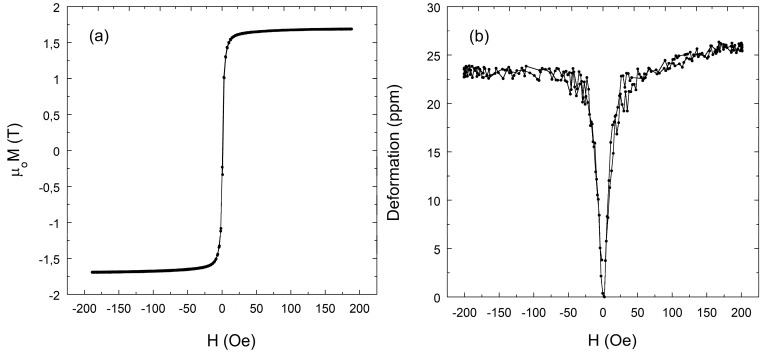
$Fe_{64}Co_{17}Si_{6.6}B_{12.4}$ composition metallic glass magnetic characterization: (**a**) hysteresis loop and (**b**) magnetostriction curve.

In the present study, Fe-based $Fe_{64}Co_{17}Si_{6.6}B_{12.4}$ composition homemade metallic glass ribbons were used. They were prepared by the single roller quenching method in the form of a long ribbon. Equal length strips (L=4 cm) were cut to perform all the magnetic and magnetoelastic characterizations. Room-temperature hysteresis loops were measured by a classical induction method, obtaining a saturation magnetization (given as internal magnetic induction in Tesla) of $\mu_{o}M_{S}\approx 1.6\;T$ and a magnetic susceptibility χ≈15,000. A magnetostriction value of $\lambda_{S}\approx 22\;\mathrm{ppm}$ was measured using strain gauges connected to an electronic Wheatstone bridge. Figure 2 shows the obtained hysteresis loop and magnetostriction curves.

### 2.2. Magnetoelastic Characterization

The metallic glasses of the present study show excellent coupling between magnetic and elastic properties, that is, the applied mechanical stress and the magnetic field generating equivalent effects in the magnetization and deformation of the materials. A direct consequence of the magnetoelastic coupling is the dependence of the elastic constants of magnetostrictive materials on the external magnetic field H, in particular the dependence of the longitudinal Young’s modulus on H, known as the ΔE effect ($\Delta E=1-E\left(H\right)/E_{S}$, with $E_{S}$ being the Young’s modulus measured at magnetic saturation (a detailed mathematical formula can be found in [21]).

This ΔE effect is easy to measure experimentally through the change in mechanical resonance as a function of the field. The resonance can be excited by an alternating field and detected by the changes in magnetic susceptibility. For this purpose, we used a home-mounted, computer-controlled magnetoelastic resonance detection apparatus [22,23] that automatically changes the DC external applied magnetic field $H_{dc}$, also known as bias field, and sweeps the frequency of the AC magnetic field $H_{ac}$ in order to drive the sample to magnetoelastic resonance at a given bias. This is the so-called resonance–antiresonance detection method. We use an HP 3589A Spectrum Analyzer in order to quickly measure the magnitude (χ) of the susceptibility curve at the magnetoelastic resonance and store the resonant frequency $f_{r}$ at the maximum and antiresonance frequency $f_{a}$ at the minimum signals, together with the signal amplitude at the resonance. Besides the susceptibility χ, we also measure its real (χ') and imaginary (χ'') components separately with the help of a Signal Recovery 7280 Lock-in Amplifier. In these measurements, we extract the frequencies $f{'}_{M}$ and $f_{m}^{'}$, at the maximum and the minimum signal of the χ', respectively, and to the frequency $f'{'}_{M}$ at maximum χ''.

All these measured frequencies and in particular the resonance ($f_{r}$) vary with the bias field $H_{dc}$, and so does the Young’s modulus, determined as $E\left(H\right)={\left[2Lf_{r}^{2}\left(H\right)\right]}^{2}\rho$ [24], where L and ρ are the length and density, respectively, of the sample. Other useful magnetoelastic parameters that can be determined from these measurements are the magnetoelastic coupling coefficient ($k=\sqrt{\left(\pi^{2}/8\right)\left(1-{\left(f_{r}/f_{a}\right)}^{2}\right)}$) [25] and the quality factor of the resonance Q. All such quantities are a function of the applied external magnetic field.

**Figure 3:**
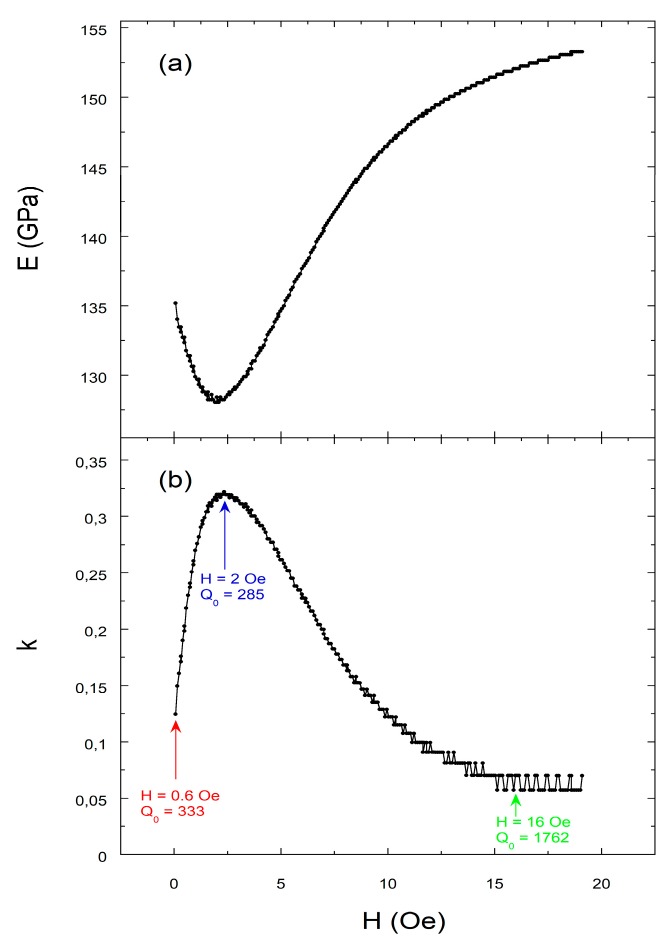
Magnetoelastic characterization of *Fe*_64_*Co*_17_*Si*_6.6_*B*_12.4_ metallic glass: magnetic field dependence of (**a**) Young’s modulus E(H); (**b**) magnetoelastic coupling coefficient k(H).

Figure 3 shows the typical external applied magnetic field dependence of Young’s modulus E(H) and magnetoelastic coupling coefficient k(H) for our magnetostrictive material. It can be seen that there is a minimum in the E(H) curve that happens at a value of the applied external magnetic corresponding to $H_{k}$ or effective anisotropy field of the sample. In the same field, the maximum of k(H) and minimum of Q(H) occur. This is an expected behavior since in fact the k value is high when the difference between $f_{r}$ and $f_{a}$ is also high, that is, the resonance curve is broad, and so its corresponding Q value (quality of the resonance curve) is poor. While the maximum of the coupling value k guarantees the best sensitivity S of a magnetoelastic resonator working as a biological or chemical sensor [13], the simultaneous occurrence of the worst Q value jeopardizes the accurate determination of the magnetoelastic resonance frequency. Bearing this in mind, we will study the Q factor under external bias field conditions for poor (k=0.065 at 16 Oe), medium (k=0.176 at 0.6 Oe), and good (k=0.282 at 2 Oe) magnetoelastic coupling, aiming to establish the most adequate method (analysis of the real and imaginary part of the susceptibility, numerical fitting of modulus of the susceptibility, and analytical calculations) to determine the quality factor of a magnetoelastic resonance.

## 3. Results: Determination of the Q Quality Factor Value

### 3.1. Full Susceptibility Curve Analysis at Resonance

**Figure 4:**
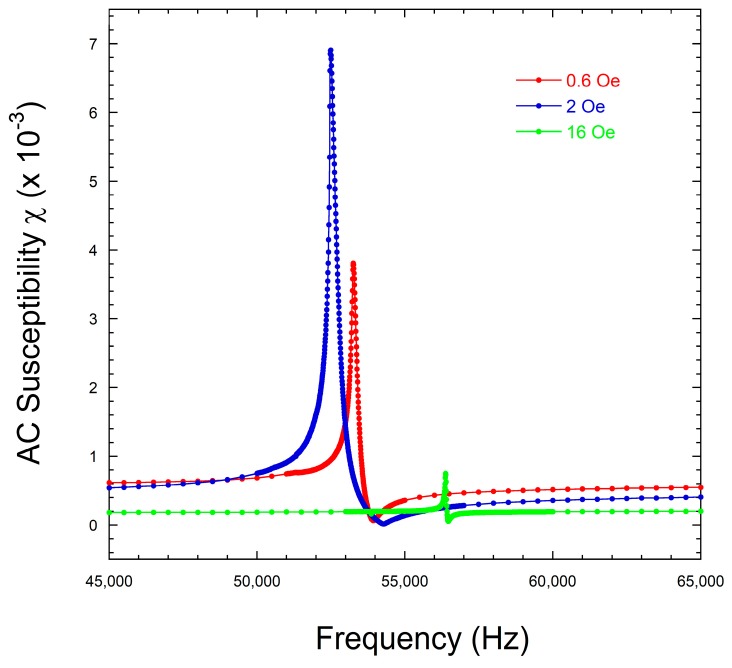
Susceptibility modulus (χ) measured for the $Fe_{64}Co_{17}Si_{6.6}B_{12.4}$ (L=4 cm) composition metallic glass, for all the bias magnetic field cases.

Figure 4 shows the magnetic susceptibility modulus (χ) curves for our $Fe_{64}Co_{17}Si_{6.6}B_{12.4}$ (L=4 cm) composition metallic glass, measured around the magnetoelastic resonance frequency for all the applied magnetic field cases under study. In these measurements, the frequency step between consecutive points was 10 Hz.

From these susceptibility modulus (χ) curves, we can directly extract the resonance $\left(f_{r}\right)$ and antiresonance $\left(f_{a}\right)$ frequencies and so estimate the magnetoelastic coupling coefficient k and the quality factor Q, from Equation (2), as Table 1 summarizes. As mentioned before, however, the values of $Q_{0}$ are quite inaccurate.

**Figure 5:**
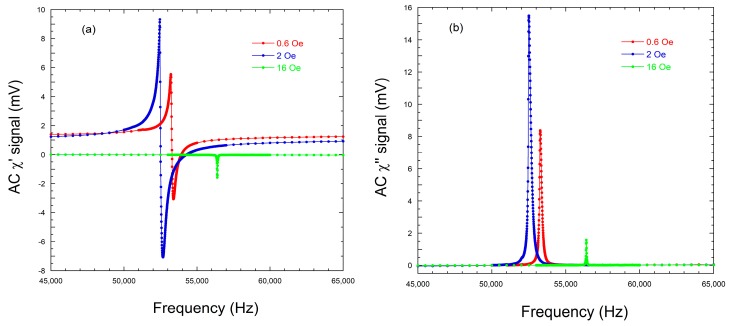
(**a**) Real, χ', and (**b**) imaginary, χ'', parts of the magnetic susceptibility for the $Fe_{64}Co_{17}Si_{6.6}B_{12.4}$ (L=4 cm) metallic glass ribbon, for the three bias magnetic field cases under study.

However, a careful measurement of those magnetoelastic resonance curves, using a Lock-in Amplifier, allows us to record the susceptibility real and imaginary parts $(\chi^{\prime }$ and $\chi^{\prime \prime }$, respectively ) as Figure 5 shows.

With the measured frequencies corresponding to maximum and minimum values of χ' ($f_{M}^{'}$ and $f_{m}^{'}$, respectively, see Figure 5a), we can use the first approximated expression often used to give an accurate value of this Q quality factor [20]:

(3) $$Q_{1}\approx \frac{f_{r}}{{f^{\prime }}_{m}-{f^{\prime }}_{M}}\;.$$

Table 2 summarizes the experimentally obtained frequency data for maximum and minimum values of χ' ($f_{M}^{'}$ and $f_{m}^{'}$, respectively) and calculated $Q_{1}$ values from Equation (3), as well as the relative difference between $Q_{0}$ and $Q_{1}$.

It must be noted that to separately obtain the real and imaginary parts of the susceptibility is a quite difficult and time-consuming experimental task. Therefore, a method to obtain Q based only on the magnitude or modulus of the susceptibility is highly desirable, though it demands more complex numerical treatment afterwards.

### 3.2. Numerical Fitting of the Magnitude of the Susceptibility Curve

**Figure 6:**
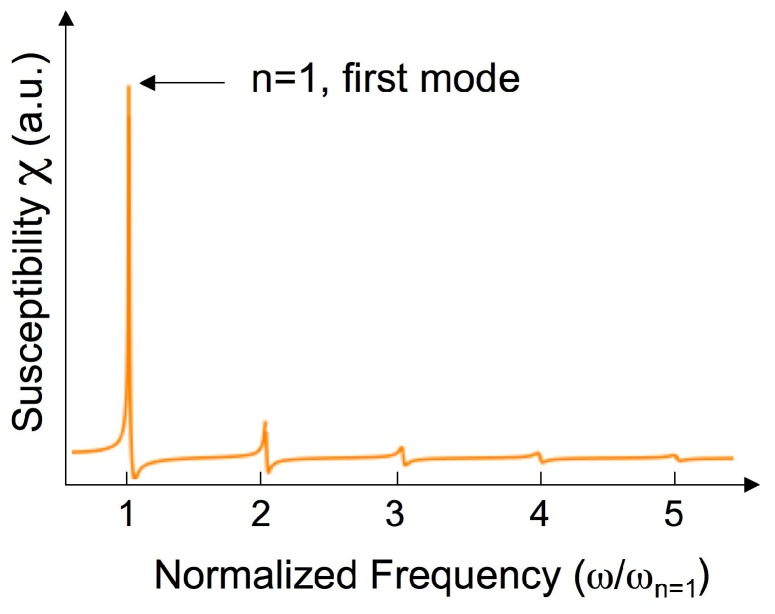
Calculated magnetic susceptibility vs. frequency behavior for a magnetoelastic ribbon, up to the fifth harmonic, using Equation (4).

In 1978 Savage et al. [25] derived the following expression for the susceptibility around the magnetoelastic resonance in a free-standing cylinder-shaped sample:

(4) $$\chi \left(\omega \right)=\chi_{0}\left[1-\frac{8k^{2}}{\pi^{2}}\underset{n}{\sum }\frac{1}{n^{2}}\times \frac{1}{1-\frac{\omega_{n}^{2}}{\omega^{2}}+iQ^{-1}\frac{\omega_{n}}{\omega }}\right],$$

where k is the magnetoelastic coupling coefficient, $\omega_{n}=2\pi f_{n}$ is the frequency of the *n*th harmonic of the excited fundamental mode (n=1), $Q^{-1}$ is a phenomenological damping coefficient, and $\chi_{0}$ is the magnetic susceptibility measured at a frequency far below the resonance [26]. Equation (4) also applies to rectangular section ribbons with a proper choice of the k factor. Figure 6 shows an example of the calculated magnetic susceptibility, up to the fifth harmonic, by using Equation (4).

A different approach to estimate Q of a magnetoelastic resonance curve is the numerical fitting of the modulus or magnitude of the experimental susceptibility around its first resonant mode (n=1) by using Equation (4). Thus, we proceed to perform numerical fittings using Mathematica© software (v.11.0), following two different strategies: (a) by using the measured $f_{r},\;f_{a},\;\chi_{0}$ values as fixed parameters and (b) by leaving these parameters to vary around the experimentally obtained ones. In both cases, our goal is to search for the optimum Q value that minimizes the $L^{2}$ *norm* between the fit and experimental data. We define such a *norm* (also called *residual*) as: ℛ $=\frac{1}{N}\underset{i=1,N}{\sum }{\left(\frac{\chi_{exp,i}-\chi_{fit,i}}{\chi_{max}}\right)}^{2}$, where $\chi_{max}=\max\left(\chi_{max,exp},\chi_{max,fit}\right)$ and N is the number of experimental points. In our measurements, N=397, 691 and 639 for H=0.6, 2 and 16 Oe, respectively. With such a definition, 0≤R ≤1, and a value of R close to 0 means very good fitting.

#### 3.2.1. Numerical Fitting of the Susceptibility Curve Using Fixed Parameters

**Figure 7:**
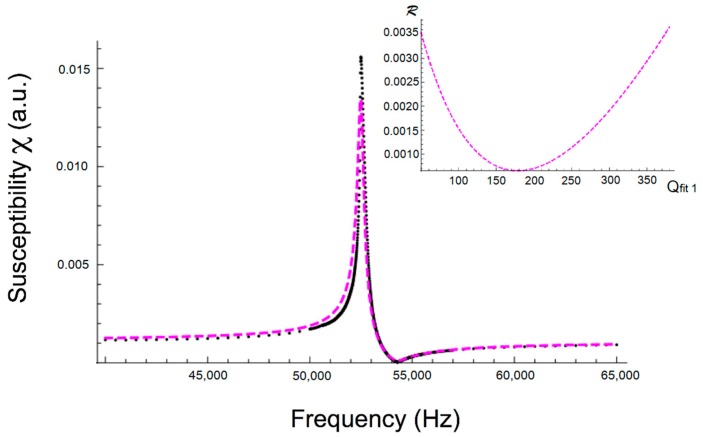
Measured resonance curve at H=2 Oe (black dots) and fitted one (magenta marks). The inset shows the *residual* ℛ change versus Q values, all obtained in calculations using procedure a.

Figure 7 shows the measured magnetoelastic resonance curve at H=2 Oe and the fitted one when procedure a) is used. The only free parameter was the quality factor Q with an initial value of $Q_{0}=285$. The sweep range for Q was 50–380, and the optimum fitting was found for $Q_{fit1}=178$ (see Figure 7 inset). For all the fits performed, the frequency step between consecutive points will be 1 Hz. The fit of Figure 7 is the best one obtained by following the a procedure. It looks satisfactory, but can still be improved.

Table 3 summarizes the results obtained for the three different magnetic field cases. While all obtained $Q_{fit1}$ values are lower than the previous $Q_{0}$ ones, it is noticeable that the worst fit corresponds to the applied magnetic field, where magnetoelastic coupling is maximum but quality factor is minimum.

#### 3.2.2. Numerical Fitting of the Susceptibility by Leaving All Parameters Free

**Figure 8:**
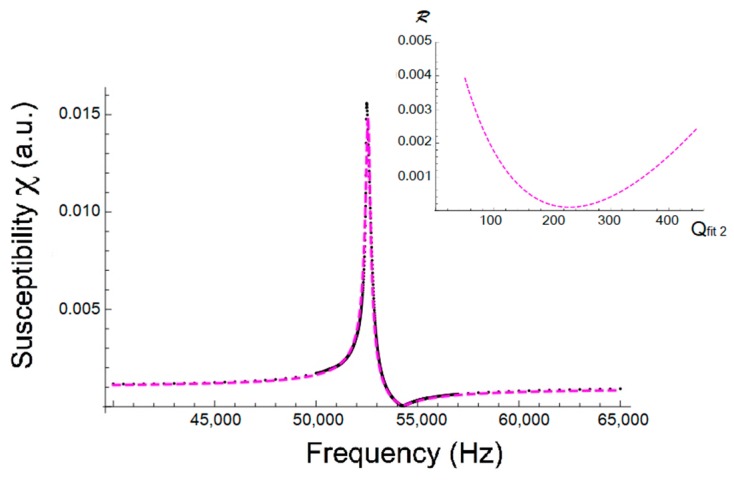
Measured resonance curve at H=2 Oe (black dots) and fitted one (magenta marks). The inset shows the *residual* ℛ change versus Q values, all obtained in calculations when using procedure b.

Figure 8 shows the measured magnetoelastic resonance curve at H=2 Oe and the fitted one when this second procedure is used: all parameters were left free in a range around the starting guess given by the experimental values appearing in Table 1. In this case we swept the Q value in the range 50–440, finding the best fit for $Q_{fit2}=229$, for the case of applied magnetic field H=2 Oe (see Figure 8 inset). Now the fit has greatly improved, as the *norm* values are much lower, especially for the curve with the highest Q value curve (at H=16 Oe) (see Table 4).

Table 4 summarizes the results obtained for the three applied magnetic field cases. Again, as with the first simulation procedure, all obtained $Q_{fit2}$ values are lower than the previous $Q_{0}$ ones and the worst fit corresponds to the applied magnetic field where magnetoelastic coupling is maximal but the quality factor is minimal.

### 3.3. An Exact Expression for the Q Factor

Starting with Equation (4) and taking into account the shape of the $\chi^{\prime }\left(\omega \right)$ curve, one of us (J.G.) has derive an exact analytical expression for the Q factor value calculation. Since magnetic susceptibility χ can be described by Equation (4) as a complex number, χ=χ'+iχ'', we can separate the real and imaginary parts of this complex expression (taking into account only the first harmonic, n=1) as follows:

(5) $$\frac{\chi '}{\chi_{0}}=1-\frac{8k^{2}}{\pi^{2}}\frac{\omega^{2}\left(\omega^{2}-{\omega_{1}}^{2}\right)}{{\left(\omega^{2}-{\omega_{1}}^{2}\right)}^{2}+{\left(\frac{\omega \omega_{1}}{Q}\right)}^{2}}\;,$$

and

(6) $$\frac{\chi ''}{\chi_{0}}=\frac{8k^{2}}{\pi^{2}}\frac{\frac{\omega^{3}\omega_{1}}{Q}}{{\left(\omega^{2}-{\omega_{1}}^{2}\right)}^{2}+{\left(\frac{\omega \omega_{1}}{Q}\right)}^{2}}\;.$$

The real part shows resonance at its maximum: ($\omega_{M}^{'}$) and antiresonance at its minimum: ($\omega {’}_{m}$) (see Figure 5a). It is well known that at the local maxima and minima, the derivative vanishes so we can apply this criteria for the frequencies $\omega =\omega_{M}^{'}$ and $=\omega {‘}_{m}$:

(7) $$\frac{d}{d\omega }\left(\frac{\chi '}{\chi^{\sigma }}\right)=0=>\frac{d}{d\omega }\frac{\omega^{2}\left(\omega^{2}-{\omega_{1}}^{2}\right)}{{\left(\omega^{2}-{\omega_{1}}^{2}\right)}^{2}+{\left(\frac{\omega \omega_{1}}{Q}\right)}^{2}}=0\;.$$

After a short calculation, and taking into account that Q>0 and single-valued (which means $\omega_{M}<\omega_{r}<\omega_{m}$), the Q factor value can be written in the following two ways:

(8) $$Q=\frac{{\omega_{m}}^{2}}{{\omega_{m}}^{2}-{\omega_{1}}^{2}}\;\mathrm{and}\;Q=\frac{{\omega_{M}}^{2}}{{\omega_{1}}^{2}-{\omega_{M}}^{2}}\;.$$

Solving these two expressions, first for $\omega_{M}=\omega_{r}$ and afterwards for Q, we finally get an exact analytical expression for the Q factor value:

(9) $$Q_{cal}=\frac{\omega_{m}^{2}+\omega_{M}^{2}}{\omega_{m}^{2}-\omega_{M}^{2}}=\frac{1+u^{2}}{1-u^{2}\;},$$

where $u=\omega {'}_{M}/\omega {'}_{m}=f{'}_{M}/f{'}_{m}$. Table 5 summarizes the experimentally measured values for those frequencies and the subsequently obtained $Q_{cal}$ values.

## 4. Discussion

All the Q values obtained by the different calculation procedures explained in this study are summarized in Table 6, while Table 7 shows the estimated errors (in %, calculated as $Error\;\left(\%\right)=\left|\frac{Q_{1}-Q}{Q_{1}}\right|\cdot 100$) among those obtained Q values. The first thing to notice is that in all cases (or calculation procedures) the higher the estimated Q value, the higher the error in its determination, and this always corresponds to the highest applied field. This is a direct consequence of the sharpness of the measured resonance curves, as well as of its low amplitude (magnetic susceptibility), as can be seen in Figure 5a. As predicted previously by other authors [20], this estimated error (if the classical $Q_{0}$ definition is used) can be as high as 20%. Surprisingly, the obtained *norm* of the fitting for this high applied field case is the lowest, this is, the numerical fits are the best for this case.

We also found that the values obtained for $Q_{1}$ (the value given by Kaczkowski [20]) and $Q_{cal}$ (exact analytical expression) are almost equal. While Kaczkowski’s expression was an approximation obtained graphically from the impedance circle of an electrical circuit, our exact formula comes from the analysis of the real part of the magnetic susceptibility around the magnetoelastic resonance.

So, if we only have the possibility of measuring or working with the magnetic susceptibility modulus, numerical fitting of the measured magnetoelastic resonance curve is needed. From the results of the numerical fittings used (as can be observed in Figure 7 and Figure 8 and Table 7), it is clear that the second procedure (leaving all parameters $f_{r},\;f_{a}$ and $\chi_{0}$ free) leads to a much better result than the first one, as deduced from the obtained lowest *norm* values for procedure b. That is, we can affirm that the $Q_{fit2}$ value obtained by using fitting procedure b can be considered the best approximation of the true Q quality factor of the magnetoelastic resonance curve.

On the other hand, if we compare the Q values obtained from numerical fits with the approximated $Q_{1}$ value given by Kaczkowski or with the $Q_{cal}$ value, the estimated error when using fitting procedure a is always higher than 20%, while for fitting (procedure b) the range is only approximately 2–6%. Thus, $Q_{1}$ or $Q_{cal}$ can be taken as approximated initial values when performing a numerical fit in order to get the most accurate Q value of a susceptibility magnetoelastic resonance curve.

It is also noticeable that Q values obtained with fitting (procedure b) are systematically lower than $Q_{1}$ or $Q_{cal}$, but this is a fact that should be expected: Equation (9) gives us the exact quality factor Q of the real part of the magnetic susceptibility around the frequency at which a magnetoelastic resonance happens, with this real part being a sharper curve than the corresponding measured susceptibility modulus. As is already well known, the sharper the curve, the higher the quality factor value. Finally, from the obtained error values, we can affirm that when using a magnetoelastic resonant platform for biological or chemical detection purposes, it is convenient to apply a bias field in the range $0<H_{bias}<H_{k}$, searching for a compromise between moderate magnetoelastic coupling and low enough error in Q value determination.

## 5. Conclusions

We have presented an extensive study of the determination of the Q factor of a magnetoelastic resonance curve. This type of resonance is of great interest in order to fabricate devices for biological or chemical detection purposes. The use of the numerical fitting of the magnetic susceptibility modulus around that magnetoelastic resonance turns out to be a useful tool to give accurate Q quality factor values. These differ by up to 20% compared with the Q values determined by following the classical definition. Comparison with approximated Q value given by Kaczkowski and by the exact analytical solution obtained from the real part of the measured susceptibility shows, as expected, that in these two last cases the quality factor value obtained is always slightly higher than that estimated from the numerical fitting. This is a direct consequence of the fact that, while the numerical fit is performed over the magnetic susceptibility modulus, Kaczkowski’s and the exact expression for have been obtained from the real part of that susceptibility curve, which is always sharper than the susceptibility modulus one.

Future work should aim to obtain an analytical expression for the Q quality factor directly obtained from the magnetic susceptibility modulus measured around the magnetoelastic resonance.

## Figures and Tables

**Table 1:** Resonance and antiresonance frequencies for $Fe_{64}Co_{17}Si_{6.6}B_{12.4}$ and calculated *k* and $Q_{0}$ values determined directly from the experimental data (this last one obtained using Equation (2)).

H (Oe)	$f_{r}\;\left(Hz\right)$	$f_{a}\;\left(Hz\right)$	Δf	k	$Q_{0}$
0.6	53,260	53,940	160	0.176	333
2	52,496	54,280	184	0.282	285
16	56,380	56,476	32	0.065	1762

**Table 2:** Frequencies for the maximum and minimum of χ' ($f_{M}^{'}$ and $f_{m}^{'}$, respectively), calculated $Q_{1}$ values (using Equation (3)) and relative error between $Q_{0}$ and $Q_{1}$.

H (Oe)	$f{’}_{M}\;\left(Hz\right)$	$f{’}_{m}\;\left(Hz\right)$	$Q_{1}$	Relative Difference (%) $\left(Q_{0},Q_{1}\right)$
0.6	53,212	53,396	290	13
2	52,456	52,672	243	15
16	56,372	56,412	1410	20

**Table 3:** Q values from the fitting of the χ susceptibility modulus, using fixed experimental parameters (procedure a).

H (Oe)	$f_{r}\;\left(Hz\right)$	$f_{a}\;\left(Hz\right)$	$Q_{fit1}$	ℛ
0.6	53,260	53,940	208	0.0041
2	52,496	54,280	178	0.0039
16	56,380	56,476	1067	0.00031

**Table 4:** Obtained parameters (resonance and antiresonance frequencies and Q) from the fitting leaving all parameters free (procedure b).

H (Oe)	$f_{r}\;\left(Hz\right)$	$f_{a}\;\left(Hz\right)$	$Q_{fit2}$	ℛ
0.6	53,301	53,927	283	0.00027
2	52,566	54,296	229	0.00060
16	56,390	56,466	1321	0.000011

**Table 5:** $Fe_{64}Co_{17}Si_{6.6}B_{12.4}$ experimentally obtained data for resonance and antiresonance frequencies of the real part of the magnetic susceptibility, and calculated Q values using Equation (9).

H (Oe)	$f_{M}^{'}\;\left(Hz\right)$	$f_{m}^{'}\;\left(Hz\right)$	$Q_{cal}$
0.6	53,212	53,396	290
2	52,456	52,672	243
16	56,372	56,412	1410

**Table 6:** Q values for all the applied magnetic field cases, obtained by the different procedures shown in this study.

H (Oe)	$Q_{0}=\frac{f_{r}}{\Delta f}$	$Q_{1}\approx \frac{f_{r}}{f_{m}^{'}-f_{M}^{'}}$	$Q_{fit1}$	$Q_{fit2}$	$Q_{cal}=\frac{f_{m}^{2}+f_{M}^{2}}{f_{m}^{2}-f_{M}^{2}}$
0.6	333	290	208	283	290
2	285	243	178	229	243
16	1762	1410	1067	1321	1410

**Table 7:** Comparison and estimated errors of the Q values obtained with the different calculation procedures.

H (Oe)	$Error\;\left(\%\right)\;Respect\;to\;Q_{1}or\;Q_{cal}$		
	$Q_{0}$	$Q_{fit1}$	$Q_{fit2}$
0.6	13	28	2.4
2	15	27	5.7
16	20	24	6.3
